# The roles of folate, MTHFR genetics, vitamin B12 in pregnancy outcomes

**DOI:** 10.3389/fnut.2026.1785263

**Published:** 2026-03-04

**Authors:** Ruihua Yang, Guanghui Li

**Affiliations:** 1 Department of Nutrition, Endocrinology, and Metabolism, Beijing Obstetrics and Gynecology Hospital, Capital Medical University, Beijing, China; 2 Beijing Maternal and Child Health Care Hospital, Beijing, China

**Keywords:** folate, Hcy, MTHFR, pregnancy outcomes, vitamin B12

## Abstract

Folate is essential for fetal development, and periconceptional folic acid (FA) supplementation is well-established for preventing neural tube defects. However, evidence regarding its role in other pregnancy outcomes, such as gestational diabetes mellitus, hypertensive disorders of pregnancy, fetal growth, miscarriage, and preterm birth, remains inconsistent. Current knowledge indicates that the effects of FA are not uniform but significantly influenced by the timing, dose, and duration of supplementation, frequently exhibiting U-shaped or timing-dependent relationships. Furthermore, methylenetetrahydrofolate reductase (MTHFR) genetic polymorphisms and vitamin B12 levels are critical modifiers of folate metabolism and its association with pregnancy outcomes. Crucially, there is a lack of quantitative studies linking circulating folate levels to the risk of adverse outcomes, and no optimal threshold range has been established to balance the prevention of different complications. This review consolidates the existing evidence on the associations between FA supplementation, circulating folate levels, and non-structural pregnancy outcomes, while elucidating the modulating roles of MTHFR genetics and vitamin B12. Besides, it highlights possible underlying biological mechanism of hyperhomocysteinemia, alterations in DNA methylation, the presence of folate receptor antibody (FRAbs), and the direct anti-inflammatory effects of folate. This review aims to provide a foundation for a future precision nutrition strategy through individual physiological folate levels, MTHFR genetics, and vitamin B12 status.

## 1 Introduction

Folate, vitamin B9, plays a central role in fundamental biological processes essential for cellular function, including nucleotide synthesis, amino acid metabolism, and methylation reactions (1). These processes are critical for both physiological development and pathological outcomes throughout pregnancy. Its synthetic form, folic acid (FA), exerts physiological effects after being converted into active folate derivatives. FA is first converted to tetrahydrofolate (THF), which acts as the central hub. THF is then sequentially converted into key derivatives, such as 5,10-methylenetetrahydrofolate (5,10-CH_2_-THF) which is indispensable for DNA synthesis, and 5-methyltetrahydrofolate (5-MTHF) that is the primary methyl donor for the methionine cycle and methylation reactions (2).

FA supplementation during periconception is widely recommended to prevent fetal neural tube defects (NTDs) (3). However, emerging evidence suggests that the influence of FA may extend beyond NTD prevention. Studies have extensively linked FA supplementation to gestational diabetes mellitus (GDM) (4), along with other outcomes including hypertensive disorders of pregnancy (HDP) (5), fetal birth weight (6), miscarriage (7), and preterm birth (PTB) (8). Nevertheless, these associations remain inconsistent. The effects of FA appear to be influenced by the timing, dose, and duration of supplementation. For example, moderate-dose (400–800 μg/d) FA supplementation for an adequate duration may minimize the risk of GDM, whereas no supplementation, low-dose, short-term, or long-term high-dose (≥800 μg/d) use is associated with increased risk (9). The increased GDM risk associated with high-dose supplementation is primarily for initiation before and in early pregnancy. In contrast, high-dose supplementation during mid-to-late pregnancy may benefit GDM recovery (10). Regarding HDP, low-dose FA supplementation initiated post-conception provides protective effects against preeclampsia (PE). However, this protective effect diminishes or disappears with inappropriate dose or suboptimal period (11). Notably, a very high dose (4 mg/d) is ineffective for primary prevention in the general population but significantly reduce recurrence in high-risk women with a history of PE when started before pregnancy (12).

Moreover, the modifying roles of genetic factors and nutritional status further complicate the interpretation of these relationships. Polymorphisms in the methylenetetrahydrofolate reductase (MTHFR), methionine synthase (MTRR) and methionine synthase(MTR) genes can impair enzyme activity, thereby disrupting homocysteine (Hcy) metabolism (13). Evidence indicates that MTHFR 677TT genotype is associated with significantly reduced enzyme activity, low folate levels, and hyperhomocysteinemia (HHcy). While MTHFR 677CC genotype carriers may show a better response to FA, current consensus holds that standard-dose FA is equally effective at achieving recommended red blood cell (RBC) folate levels for NTD prevention across genotypes (14). Notably, the T allele of the MTHFR C677T gene polymorphism tends to increase the risk of GDM (15), PE (16, 17), and small for gestational age (SGA) infants (18). MTHFR 1298CC genotype is also significantly associated with the increased risk for PE (19, 20) and SGA (18). The underlying mechanism involves disrupting normal epigenetic control. Folate and S-adenosylmethionine (SAM) availability influence site-specific placental DNA methylation, particularly in individuals with the MTHFR C677T variant. While genome-wide methylation is generally stable, the ratio of SAM to S-adenosylhomocysteine (SAH) and RBC folate levels significantly affect CpG methylation in certain genomic regions, with a more pronounced effect in MTHFR 677TT genotype carriers (21). Folate metabolism is intricately linked to vitamin B12, a critical cofactor for methionine synthase. Consequently, vitamin B12 deficiency or an altered folate:vitamin B12 ratio can compromise folate’s metabolic balance, and is thus related to pregnancy outcomes (22). For instance, the combination of high folate with low vitamin B12 levels shows the strongest association with elevated GDM risk (23), while a low folate:vitamin B12 ratio is a significant risk factor for PE (24). The Hcy demethylation pathway is influenced by folate, vitamin B12, and MTHFR gene polymorphism. HHcy serves as a key convergence point for the interaction between genetic and nutritional factors. The endothelial and placental dysfunction induced by HHcy is likely a core mechanism linking abnormal folate metabolism with adverse pregnancy outcomes (25). Other potential mechanisms may include alterations in DNA methylation (26), the presence of folate receptor antibody (FRAbs) (27), and the anti-inflammatory effects of folate itself (28), are also involved. These mechanisms may be intertwined and collectively contribute to the disease pathogenesis.

World Health Organization (WHO) recommends that women supplement with 400 μg of FA daily, starting at least 3 months before conception and continuing until the first 12 weeks of pregnancy, to prevent NTDs (29). However, evidence regarding the effect of FA on other pregnancy outcomes remains conflicting. The recommended upper FA intake levels range from 200 to 1,000 μg/d by age (30). Notably, current evidence on FA supplementation primarily derives from studies focusing on supplementation dose and duration. The observed variability in individual response indicates that a fixed supplement dose does not guarantee uniform circulating folate levels due to differences in genetics, metabolism, and baseline nutritional status. Therefore, FA supplementation should be personalized based on the monitoring of circulating folate levels. Serum or plasma folate primarily reflects recent folate intake and metabolism, significantly influenced by factors like diet and medication. In contrast, RBC folate is a sensitive indicator of long-term folate status, reflecting folate levels during the preceding 120 d (i.e., the half-life of RBCs). Moreover, RBC folate parallels liver concentrations (accounting for ~50% of total body folate) and is thus considered to reflect tissue folate stores (31). WHO recommends a RBC folate >906 nmol/L for women to prevent NTDs (29), while no definitive threshold has been established for serum or plasma folate. Critically, there is still a lack of an optimal folate threshold range that balances NTD prevention and minimizing the risk of other adverse pregnancy outcomes. Therefore, the critical next step is to establish a quantitative link between circulating folate (especially RBC folate) levels and adverse pregnancy outcomes. Such research would shift the paradigm from simply recommending a standard dose to defining a target physiologic range. The dose- and duration-dependent response of FA supplementation could then be personalized, guided by the objective of achieving and maintaining an individual’s circulating folate within that protective range. In this framework, monitoring circulating folate serves as the essential tool for personalizing and optimizing supplementation strategies. A multicenter double-blind randomized controlled trial (RCT) showed that 4.0 mg FA supplementation is not associated with a different occurrence of congenital malformations compared to 0.4 mg supplementation. However, 4.0 mg FA supplementation is associated with a lower occurrence of other adverse pregnancy outcomes (32).

This review evaluates the associations between FA supplementation (including timing, dose, and duration), circulating folate levels, and various non-structural pregnancy outcomes such as GDM, HDP, fetal birth weight, abortion, and PTB. We further examine the modulating roles of MTHFR gene polymorphisms and vitamins B12 to elucidate the heterogeneity in existing and integrates the underlying biological mechanisms. Based on the consolidated evidence, this review aims to provide insights for future strategies for precision nutrition and clinical practice.

## 2 Folate, MTHFR genetics, vitamin B12 and pregnancy outcomes

### 2.1 GDM

#### 2.1.1 Timing-, dose- and duration- dependent response of FA supplementation

The associations between FA supplementation and GDM risk are highly complex. A longitudinal study of 24,429 Chinese women found that FA supplementation in early pregnancy was inversely associated with GDM (OR = 0.75, 95%CI: 0.63–0.90), particularly in women with normal pre-pregnancy body mass index (BMI) (4). In contrast, two Nordic cohort studies involving 791,709 Norwegian and 1,112,817 Swedish pregnancies also demonstrated that prescribed high-dose FA supplementation (1–5 mg/d) before pregnancy or in early pregnancy was associated with an increased GDM risk (Norway: OR = 1.33, 95%CI: 1.15–1.53; Sweden: OR = 1.56, 95%CI: 1.41–1.74) (33). Interestingly, Zou et al. described a U-shaped relationship between FA supplementation before 24–28 gestational weeks and GDM risk. Compared to moderate intake (400–799 μg/d) for an adequate duration (>1 month before pregnancy and >3 months during pregnancy), no supplementation, low-dose (< 400 μg/d) or short-term(< 1 month), and high-dose (≥800 μg/d) supplementation for an adequate duration were associated with a 228, 28, and 188% increased risk of GDM, these associations were stronger in women with pre-pregnancy BMI ≥ 25 kg/m^2^ (9). A prospective cohort study in China (*n* = 4,353) also found that high-dose FA (≥800 μg/d) for a long duration [continuous at least 4 weeks pre-pregnancy and continued for at least 16 weeks during pregnancy before oral glucose tolerance test (OGTT)] significantly increased GDM risk (OR = 2.09, 95%CI:1.30–3.36), particularly elevating postprandial glucose. Lower doses (400–800 μg/d) or shorter durations showed no significant association, supporting 400 μg/d as the optimal dose unless medically indicated (34). Further research highlighted the timing-dependent risk. A meta-analysis of 26 datasets from 13 studies showed that longer duration (≥3 months) of FA supplementation conferred significantly higher GDM risks (OR = 1.56, 95%CI: 1.02–2.39) (35), especially before pregnancy (36). In contrast, continuous supplementation during the middle and late pregnancy may exert potential beneficial effects. An open-label interventional study (*n* = 254) demonstrated that high-dose FA (800 μg/d) during mid-late pregnancy accelerated GDM recovery in MTHFR 677TT genotype carriers by 27 days compared to standard dose (400 μg/d). Concomitantly, the rate of GDM recovery increased by 24.7% at 100 days of treatment with the standard-dose group as reference (10).

#### 2.1.2 Circulating folate levels

Maternal circulating folate levels have been shown to be associated with GDM risk. However, existing studies have yielded inconsistent conclusions. The comprehensive meta-analysis of 20 studies concluded that serum folate levels in the GDM group were significantly higher than in the non-GDM group only in the second trimester (13–28 gestational weeks). RBC folate levels in the GDM group were significantly higher both in the first (1–12 gestational weeks) and second trimesters (13–28 gestational weeks), indicating that high serum and RBC folate levels increase the risk of pregnant women developing GDM (37). This aligns with a meta-analysis of 26 datasets from 13 studies, which reported a 96% increased GDM risk with high RBC folate (OR = 1.96, 95%CI: 1.48–2.61) and a 23% increase with high plasma folate (OR = 1.23, 95%CI: 1.02–1.48). The dose–response analysis revealed that each 200 ng/mL increase in RBC folate was significantly associated with 8% higher GDM risk (35). A retrospective case–control study found that high serum folate (OR = 1.84, 95%CI: 1.07–3.16) significantly increased GDM risk, particularly in mothers aged < 30 years and those with a pre-pregnancy BMI < 24 kg/m^2^ (38). In contrast, a systematic review and meta-analysis of 12 studies concluded that while a few studies linked higher RBC folate levels to an increased GDM risk, the majority of evidence suggested no association between serum folate and GDM (39). Overall, more consistent evidence links elevated RBC folate to higher GDM risk, in contrast to the contradictory findings for serum or plasma folate. This may be attributed to the fact that RBC folate reflects long-term folate status, which is more physiologically stable (40). Beyond total folate levels, emerging research further elucidates the distinct roles of specific folate metabolites in GDM development. Elevated RBC UMFA levels (OR = 1.82, 95%CI: 1.23–2.69) in early pregnancy, and high RBC 5-MTHF levels (OR = 1.48, 95%CI: 1.10–2.00) in middle pregnancy, were significantly associated with an increased risk of GDM (41).

Although some studies have explored the threshold for circulating folate, no unified range has been established to guide clinical practice. For serum folate, retrospective studies in Chinese populations suggest an association between elevated levels and increased GDM risk, though proposed thresholds vary. A study of 27,128 women identified a risk threshold at >32.5 nmol/L (14.35 ng/mL) in early pregnancy (42). Another described a J-shaped relationship, with risk increasing beyond 43.10 nmol/L (19.02 ng/mL) (38). A large cohort study (*n* = 42,478) further indicated that levels >45.3 nmol/L (20 ng/mL) before 24 gestational weeks were associated with higher GDM incidence (13.9% vs. 10.2%) and elevated OGTT glucose (43). More consistently, for RBC folate, Chen et al. reported RBC folate ≥ 1,360 nmol/L (600 ng/mL) in early pregnancy increased 58% GDM risk (44). A meta-analysis of 12 studies also reported that RBC folate ≥ 1,360 nmol/L (600 ng/mL) increased GDM risk (OR = 2.16, 95%CI: 1.70–2.74) (45). Thus, an RBC folate level of 1,360 nmol/L (600 ng/mL) may represent a potential threshold for preventing the onset of GDM, though further validation is required to confirm its clinical relevance and establish causality.

#### 2.1.3 MTHFR genetics modulation

The relationship between these MTHFR polymorphisms and GDM remains complex and population-specific. Several large cohorts from diverse regions, such as Vietnam (46), Canada (47), and South Africa (48), found no significant link between MTHFR C677T gene polymorphisms and GDM risk. However, a meta-analysis suggested that the T allele of the MTHFR C677T gene polymorphism tended to increase GDM susceptibility, especially for Asians, whereas the MTHFR A1298C gene polymorphism was not associated with an increased risk of this metabolic disorder of pregnancy (15). Moreover, the MTHFR C677T gene polymorphism is also linked to insulin levels, suggesting a metabolic influence (48, 49). Specifically, the MTHFR 677 T allele was associated with higher fasting insulin concentrations only in GDM women (48). In diabetes patients, the MTHFR CC genotype was associated with higher serum insulin levels (*β* = 0.15, *p* < 0.01) and higher HOMA-IR (*β* = 0.15, *p* < 0.01) (49). Another meta-analysis of 17 studies focusing on 12,345 Chinese populations also indicated that TT genotype increased the risk of GDM by 124% compared to CC genotype (50). Furthermore, Liu et al. found that the MTHFR C667T TT genotype increased early-onset GDM risk in Chinese pregnant women (OR = 4.00, 95%CI: 1.24–12.86) (51). However, the CC genotype is also linked to an elevated GDM risk through a distinct and indirect pathway, highlighting the complex and multifactorial etiology of GDM. Specifically, the MTHFR 677CC genotype was also associated with higher serum folate, indirectly increased GDM risk via elevated glucose levels in Chinese Han pregnant women (52). Notably, the effect of abnormal BMI on the risk of GDM is modulated by the MTHFR genotype. A study of 5,614 mother-fetus pairs found that underweight was associated with a decreased risk of GDM in women with the MTHFR 1298AA or 677CC genotype. Conversely, overweight or obesity was associated with an increased risk of GDM in MTHFR 1298 AC + CC or 677CT + TT genotype (53). Beyond MTHFR gene polymorphisms, variants in other folate-metabolism genes such as methylenetetrahydrofolate dehydrogenase 1 (MTHFD1) have also been implicated in GDM risk, underscoring the multifaceted genetic and metabolic basis of GDM pathogenesis. A case–control study found that carrying at least one A allele (either the AA or GA genotype) of MTHFD1 G1958A polymorphism was significantly associated with a reduced risk of GDM (OR = 0.5778, 95%CI: 0.3542–0.9412, *p* = 0.031), highlighting the role of folate metabolism in GDM pathogenesis (54). Currently, direct evidence on the association between the MTRR and MTR gene polymorphisms and the risk of GDM remains limited. Available studies have not identified a significant link between these genetic variants and GDM susceptibility.

#### 2.1.4 Vitamin B12 modulation

A retrospective cohort study of 42,478 pregnant women in China found a significantly higher serum folate levels before 24 gestational weeks and an increased risk of GDM, independent of vitamin B12 status (43). A meta-analysis of 13 observational studies also demonstrated that no significant association was found for vitamin B12 (35). More research has supported that the combination of high folate with low vitamin B12 levels showed the strongest association with elevated GDM risk (23). A meta-analysis of 15 studies found that maternal vitamin B12 levels deficiency in later pregnancy was associated with a 59% increase in GDM risk, and the combination of high folate and low vitamin B12 showed the strongest association (OR = 1.87, 95%CI: 1.46–2.41). These effects were particularly significant in Asian populations (55). Other studies supported that maternal vitamin B12 deficiency significantly increased GDM risk (OR = 1.46, 95%CI: 1.21–1.79) (56), and concurrent high RBC folate and vitamin B12 deficiency significantly amplified GDM risk (OR = 2.40, 95%CI: 1.60–3.61) (45). Further studies revealed that the rate of vitamin B12 insufficiency was higher than folate deficiency [42.3% vs. 1.3% in Netherlands (57); 37.4% vs. 4.6% in UK (58)]. Saravanan et al. showed that higher B12 was associated with a 14.4% lower risk of GDM, while higher folate was associated with an 11% increased risk. The combination of low B12 and high folate was associated with a 74.2% increase in GDM risk (57). These findings are consistent with a nested case–control study, which found that in early pregnancy, an elevated folate-to-vitamin B12 ratio was associated with increased GDM risk (OR = 1.67, 95%CI: 1.08–2.56) (59). Beyond vitamin B12, other B vitamin such as vitamin B6 has also been implicated in GDM. A longitudinal metabolomic study found that vitamin B6 metabolism was significantly enriched in GDM pathways across multiple trimesters, specifically highlighting disturbances in vitamin B6 metabolism in relation to GDM progression (60). Wang et al. further found that vitamin B12 levels >150 pmol/L were protective against GDM, and demonstrated nonlinear dose–response relationships for serum vitamin B6 with GDM risk (61).

In summary, (1) Supplementation strategy: Moderate-dose supplementation (e.g., 400 μg/d) initiated in early pregnancy and continued through mid-to-late gestation may be beneficial or risk-neutral, whereas long-term pre-pregnancy or high-dose (≥800 μg/d) supplementation in early pregnancy significantly increases GDM risk, suggesting the existence of a time-based therapeutic window and a dose threshold. (2) Circulating biomarkers: The association between RBC folate levels and increased GDM risk is more consistent, with a potential alert threshold around 1,360 nmol/L (600 ng/mL); findings for serum/plasma folate remain conflicting. (3) Genetic modulation: MTHFR gene polymorphisms can modify the effect of traditional risk factors like BMI on GDM susceptibility. (4) Nutritional modulation: A high folate–low vitamin B12 imbalance is a risk factor for GDM.

### 2.2 Folate, MTHFR genetics, vitamin B12 and HDP

#### 2.2.1 Timing-, dose- and duration- dependent response of FA supplementation

The association between FA supplementation and HDP remains controversial, with a more significant association observed for PE than for gestational hypertension (GH). This may stem from PE’s core features of placental insufficiency and vascular endothelial dysfunction, in which folate-mediated one-carbon metabolism may play a more direct role by influencing endothelial function and oxidative stress. In contrast, GH may primarily represent a hemodynamic adaptation to placental ischemia with less pronounced systemic vascular injury (62). Wen et al. found that FA-containing multivitamins reduced PE risk (RR = 0.37, 95%CI: 0.18–0.75) (5). A systematic review and meta-analysis of 20 studies (*n* = 359,041) demonstrated that low-dose FA supplementation was associated with a significant 17% reduction in the risk of PE, particularly when initiated post-conception (31% reduction) or in women with pre-pregnancy BMI < 25 kg/m^2^ (32% reduction). No significant association was observed with GH (11). A prospective cohort study of 3,647 Chinese women also found that FA supplementation in early pregnancy was associated with a 40% reduced risk of PE in lean mothers (BMI < 25 kg/m^2^) (63). However, an Australian cohort study of 2,261 pregnancies found that FA supplementation in the first trimester significantly reduced PE risk (OR = 0.42, 95%CI: 0.13–0.98), with a particularly strong effect among women with BMI ≥ 25 kg/m^2^ (64). The different outcomes may stem from variations in baseline folate levels due to national fortification policies. In China, without fortification, supplementation corrected a deficiency. In Australia, with existing food fortification, supplementation may have addressed the higher metabolic demands in overweight women.

Notably, the dose and duration of FA supplementation are critical. A large cross-sectional study in China (*n* = 10,662) found that multivitamins containing 800 μg FA in early pregnancy significantly reduced PE risk (RR = 0.09, 95%CI: 0.03–0.33) compared to non-users, with the lowest incidence (0.1% in PE group vs. 1.4% in controls) (65). Women who supplemented with < 800 μg/d FA at 15-week gestation had a higher incidence of PE (10.3%) compared with women who did not supplement (6.1%) or who supplemented with ≥ 800 μg (5.4%) (20). Li et al. showed no association between FA supplementation < 800 μg/d and PE (66). A high-quality RCT including 2,301 high-risk women from 70 obstetrical centres in five countries (Argentina, Australia, Canada, Jamaica, and UK) further demonstrated very high-dose FA supplementation (4 mg/d) from 8 to 16 weeks of gestation did not prevent PE (the incidence of PE in FA supplementation group vs. placebo group: 14.8% vs. 13.5%; RR = 1.10, 95%CI: 0.90–1.34, *p* = 0.370) (67). However, for women with a history of PE, high-dose FA supplementation is protective. A RCT of 1,576 women who had PE or eclampsia in their last pregnancy demonstrated that FA supplementation with 4 mg/d initiated 3 months before pregnancy and continued throughout gestation significantly reduced the recurrence of PE in high-risk women, compared to a standard dose (0.4 mg/d) (12). Moreover, *in vivo* studies in mice revealed that oral treatment with 5-MTHF prevented and treated HDP when administered either before or during pregnancy, respectively, and normalized placental and fetal growth restriction if administered from mid-gestation onward (68).

#### 2.2.2 Circulating folate levels

Evidence regarding the association between circulating folate levels and HDP remains limited and inconsistent. A retrospective cohort study of 11,549 pregnant women showed that lower serum folate levels (< 5th percentile) were associated with higher risks of PE (OR = 1.89, 95%CI: 1.28–2.81) (24). Yadav et al. clarified that mean concentration of serum folate was lower in severe PE (35.4 ± 24.1 ng/mL) when compared with mild cases of PE (57 ± 23.4 ng/mL) (69). Another study also found that plasma folate was negatively correlated with systolic pressure (r = −0.105, *p* = 0.048) (70), and the RBC folate level below 906 nmol/L was significantly associated with a 4.86-fold higher risk of PE (71). Jing et al. established severe and moderate folate deficiency rat models by providing a folate-deficient diet from birth or weaning, respectively. Compared to controls, both deficient groups showed decreased 5-MTHF in plasma and RBC and increased plasma Hcy. However, no significant differences in sustained hypertension or proteinuria were observed among the three groups, suggesting that folate deficiency alone may not induce PE in rats (72).

#### 2.2.3 MTHFR genetics modulation

A large retrospective study of 4,246 patients identified MTRR A66G as the most prevalent gene polymorphism in PE, followed by MTHFR C677T. The frequencies of the MTRR A66G and MTHFR C677T gene polymorphisms among patients with PE were 24.5 and 9%, respectively. In contrast, the MTR A2756G mutation was rarely associated with the condition (73). Research found a higher frequency of the TT genotype and T allele of the MTHFR C677T gene polymorphism in women with PE (16, 17), implicating them as genetic risk factors, and the CT genotype also increased PE risk, while no significant difference was observed in the CC genotype (17). Furthermore, MTHFR 677 T allele carriers had significantly higher sFlt-1 serum levels, a higher median sFlt-1/PlGF ratio, and a greater likelihood of having an sFlt-1/PlGF ratio ≥ 85 (19). Notably, MTHFR 1298CC genotype was also significantly associated with increased risk for PE (19, 20). Pre-pregnancy BMI significantly interacted with MTHFR genotype to modulate risk (74). A study of 5,614 mother-fetus pairs quantified this interaction, finding that obesity group with MTHFR 1298 AC + CC (OR = 6.49, 95%CI: 2.67–15.79) and the overweight group with the 677CC genotype (OR = 4.72, 95%CI: 2.13–10.45) (53).

#### 2.2.4 Vitamin B12 modulation

A systematic review and meta-analysis showed that serum vitamin B12 levels in women with PE were significantly lower than those in healthy women (mean = −15.24 pg./mL, 95%CI: −27.52, −2.954, *p* < 0.015), but heterogeneity between studies was high (*I*^2^ = 97.8%, *p* = 0.0103) (75). Current evidence indicates that low ratio of folate and vitamin B12 levels was a significant risk factor for PE. This imbalance appears to be more critical than isolated folate deficiency or elevated vitamin B12 levels alone. A retrospective cohort study of 11,549 pregnant women in China found serum folate-to-B12 ratio < 20.47 significantly elevated PE risk (OR = 1.81, 95%CI: 1.09–3.00) (24). A low serum folate-to-vitamin B12 ratio serves as a critical biomarker indicating a disruption in one-carbon metabolism. This imbalance may arise from three main mechanisms: absolute folate deficiency, absolute vitamin B12 deficiency which traps folate in a non-functional state, or functional B12 deficiency where circulating levels appear adequate but cellular utilization is impaired (22, 76).

Overall, (1) Supplementation strategy: Low-dose FA supplementation, particularly when initiated post-conception, is associated with a reduced risk of PE. The protective effect may be more pronounced in specific subgroups (e.g., lean or overweight women, potentially dependent on baseline folate status). The efficacy of high-dose FA (≥4 mg/d) for prevention remains unconfirmed for the general population but shows benefit for women with a history of PE. (2) Circulating biomarkers: Evidence linking circulating folate levels to HDP is limited and inconsistent, though some studies suggest that severe deficiency may be associated with increased risk. (3) Genetic modulation: Polymorphisms in MTHFR (C677T, A1298C) and MTRR (A66G) genes are associated with an elevated risk of PE, and this risk is significantly modified by pre-pregnancy BMI. (4) Nutritional modulation: A low folate-to-vitamin B12 ratio emerges as a significant risk factor for PE.

### 2.3 Folate, MTHFR genetics, vitamin B12 and fetal birth weight

#### 2.3.1 Timing-, dose- and duration- dependent response of FA supplementation

A systematic review of 37 studies found that folate deficiency showed the strongest association with low birth weight (LBW) compared to other key micronutrients (6). In line with this, research shows that FA supplementation during pregnancy increases fetal birth weight. A cross-sectional study in China revealed that maternal FA supplementation during pregnancy significantly increased newborn birth weight by an average of 29.56 g (77). Another cross-sectional study of 7,318 mother-infant pairs in Northwest China found that maternal FA and dietary folate intake were related to sex-specific birth weight. Specifically, FA supplementation during pre- and post- conception was associated with a 52.8 g increase in male birth weight, and higher total folate intake (FA supplementation and dietary folate) increased birth weight in both males (*β* = 38.8, 95%CI: 5.0–72.5, *p* = 0.024) and females (*β* = 42.4, 95%CI: 6.7–78.1, *p* = 0.022) (78). Further research found that maternal FA supplementation reduced the risk of LBW and small for gestational age (SGA) infants. Guo et al. found that FA supplementation before conception and during pregnancy was associated with a reduced risk of SGA (OR = 0.72, 95%CI: 0.60–0.86). The protective effect was stronger for term SGA (≥37 weeks, OR = 0.70, 95%CI: 0.58–0.85) and among nulliparous women (OR = 0.67, 95%CI: 0.54–0.84) (79). Similarly, Yang et al. found that FA supplementation was associated with a reduced risk of LBW (OR = 0.80, 95%CI: 0.66–0.97), with stronger effects observed for term-LBW (OR = 0.59) and multiparous-LBW (OR = 0.72) (80). Neither study found a significant protective effect for dietary folate intake (79, 80).

Most studies support that 400 μg/d FA supplementation effectively improves birth outcomes. A large cohort in southern China (*n* = 200,589) demonstrated that maternal 400 μg/d FA supplementation significantly reduced the risks of LBW (RR = 0.85, 95%CI: 0.80–0.90) and SGA (RR = 0.93, 95%CI: 0.89–0.96). The incidence of LBW (2.09% vs. 2.27%) and SGA (5.73% vs. 5.90%) was lower in women who took FA compared to those who did not (81). A hospital-based study of 8,523 Chinese women and their infants found that periconceptional 400 μg/d FA supplementation reduced the risk of SGA birth by 18%, with a stronger effect observed in overweight women (OR = 0.55, 95%CI: 0.36–0.85) (82). Furthermore, the benefit is substantially greater in twin pregnancies. A cross-sectional study (*n* = 28,174) in China reported that periconceptional 400 μg/d FA supplementation reduced the risk of LBW by 15% in singletons and 55% in twins, and the risk of SGA by 18% in singletons and 50% in twins (83).

In addition to dose, the timing and duration of supplementation are also critical. Evidence consistently indicates that initiating FA supplementation before conception confers a stronger protective effect against SGA than starting after conception, with one meta-analysis reporting RRs of 0.70 versus 0.84, respectively (84). This is supported by a large cohort in China (*n* = 240,954) showing a significant risk reduction of SGA only for preconceptional supplementation (RR = 0.81, 95%CI: 0.70–0.95, *p* = 0.008), with a clear dose–response relationship based on frequency of use (8). Furthermore, longer duration is also beneficial. A cross-sectional study of 7,307 postpartum women in Northwest China found that FA supplementation >60 d (OR = 0.78, 95%CI: 0.65–0.94, *p* = 0.010) in the first trimester was significantly associated with reduced risk of SGA births compared to non-users, with each 10-day increase in supplementation reducing SGA risk by 3% (85).

However, excessive or long-term supplementation may also pose risks. A prospective birth cohort found that continuing FA supplementation (400 μg/d) after the first trimester significantly increased the risk of LGA birth (RR = 1.98, 95%CI: 1.29–3.04), but did not significantly affect the risk of SGA birth (86). Supporting a causal role of folate excess, a mouse study showed that high FA supplementation (20 mg/kg vs. 2 mg/kg) increased male offspring birth weight and induced metabolic disorders, such as elevated post-load blood glucose at 9 and 13 weeks post-weaning and increased triglyceride or cholesterol levels at 17 weeks, suggesting folate excess may promote obesity development (87). Despite these indications, quantitative studies on the association between maternal circulating folate levels and neonatal birth weight are still lacking.

#### 2.3.2 MTHFR genetics modulation

Gene-nutrient interactions are prominently observed under conditions of folate inadequacy. A prospective cohort study of 1,873 nulliparous women found significant gene-nutrient interactions between maternal FA supplementation and MTHFR gene polymorphisms on SGA risk. Non-FA supplementation users with MTHFR 1298CC genotype had significantly increased SGA risk (OR = 2.91, 95%CI: 1.52–5.60) compared to women with FA supplementation and the wild-type genotype. Similar patterns were observed for MTHFR 677 T allele carriers (OR = 1.87, 95%CI: 1.21–2.88) (88). Conversely, genetic effects on adverse pregnancy outcomes are not evident in the context of sufficient folate. In a Chinese study, the homozygote frequencies of MTHFR C677T, MTHFR A1298C, MTRR A66G, and MTR A2756G were 44.2, 1.4, 6.7, and 1.3%, respectively, and the average serum folate concentration was 11.95 ng/mL and the folate deficiency rate was 0.47%. There were no significant associations between polymorphisms in MTHFR C677T, MTHFR A1298C, MTRR A66G, or MTR A2756G and the risks of LBW and SGA (*p* > 0.05) (89). This suggests that adequate FA supplementation may compensate for the low metabolic efficiency caused by genetic variations.

Moreover, evidence indicated that certain genetic influences could persist independently of nutritional compensation, operating through more sophisticated mechanisms such as sex-specific epigenetic programming. A Spanish cohort study found that the maternal MTHFR 677CT/TT genotype was significantly associated with altered birth anthropometry, increasing the risk of SGA, depending on infant sex. Notably, these associations persisted despite adequate maternal folate intake (90). Supporting an epigenetic pathway, a case–control study further identified significantly higher DNA methylation levels at specific H19 differentially methylated region (DMR) sites (sites 7.8, 9, 17.18, *p* = 0.030, 0.016, 0.050) in SGA infants. The association was stronger in male infants whose mothers took FA around conception, suggesting sex-specific epigenetic effects of folate (91). These findings highlight that maternal genetic factors may confer sex-specific susceptibility to SGA, with altered imprinting at loci like H19 representing a plausible mediating mechanism.

#### 2.3.3 Vitamin B12 modulation

Maternal vitamin B12 and folate status are pivotal predictors of birth weight, emphasizing the crucial role of nutritional factors in influencing neonatal health outcomes (92). A systematic review reported that lower maternal vitamin B12 levels, as well as an imbalance in the vitamin B12-to-folate ratio, were significantly associated with an increased risk of LBW (22). A retrospective cohort study (*n* = 11,549) found that a higher serum folate:vitamin B12 ratio was associated with an increased birthweight (*β* = 60.99, 95%CI: 29.52–92.45) and a higher risk of LGA newborns (OR = 3.08, 95%CI: 1.63–5.83), whereas a lower ratio with a decreased birthweight (*β* = −43.81, 95%CI: −75.62, −12.00) and a lower risk of LGA newborns (OR = 0.75, 95%CI: 0.56–1.00) (24). An altered maternal folate-to-B12 ratio was significantly related to reduced newborn placental weight (PW), head circumference (HC), chest circumference (CC), and body weight (BW), as well as cord folate and vitamin B12 levels. High ratios downregulated placental one-carbon metabolism genes, including methionine synthase (MS, *p* < 0.001), glycine N-methyltransferase (GNMT, *p* < 0.05), and cystathionine-β-synthase (CBS, *p* < 0.01), and elevated Hcy (93). There was a negative correlation between cord vitamin B12 level and birth weight and head circumference (*r* = −0.21, *p* = 0.004 and *r* = −0.16, *p* = 0.036, respectively) (94). In contrast, Deepa et al. discovered that vitamin B6 levels and impaired folate status, but not vitamin B12, were associated with LBW. Specifically, vitamin B6 levels were significantly inversely associated with birth weight, and women with folate deficiency were approximately twice at risk of LBW (95).

In summary, (1) Supplementation strategy: Periconceptional and early-pregnancy FA supplementation (particularly 400 μg/d) significantly reduces the risk of LBW and SGA, with benefits extending to twin pregnancies. Initiating supplementation before conception yields the strongest protective effect. However, continuing high-dose or prolonged supplementation beyond the first trimester may increase the risk of LGA. (2) Circulating iomarkers: Quantitative studies are currently lacking. (3) Genetic modulation: MTHFR polymorphisms increase the risk of impaired fetal growth primarily under conditions of folate inadequacy. Adequate FA supplementation can effectively compensate for this genetic susceptibility, though some sex-specific epigenetic effects may persist independently. (4) Nutritional modulation: An imbalance in the folate-to-vitamin B12 ratio is associated with adverse effects on birth weight and fetal anthropometry.

### 2.4 Folate, MTHFR genetics, vitamin B12 and abortion

#### 2.4.1 Timing-, dose- and duration- dependent response of FA supplementation

Multiple observational studies have reported an association between disrupted folate metabolism and an increased risk of spontaneous abortion. A systematic review and meta-analysis of 23 studies (2052 cases vs. 1,476 controls) found significantly lower folate (serum SMD = −1.63; RBC SMD = −1.30) in patients with recurrent spontaneous abortion (RSA) (7), a finding corroborated by metabolomic studies identifying folate metabolism perturbations in miscarriage (96). A hospital-based study of 1,060 infertile women with a history of IVF/intracytoplasmic sperm injection (ICSI) failure highlighted the clinical impact by demonstrating that higher serum Hcy levels were associated with lower fertilization rates in IVF/ICSI cycles. It further demonstrated that FA supplementation intervention improved pregnancy outcomes following freeze embryo transfer (FET) in women with a history of FET failure by monitoring the reduction in Hcy levels (97). Lei et al. demonstrated that periconceptional FA supplementation (OR = 0.50, 95%CI: 0.38–0.65) and adequate FA supplementation (OR = 0.44, 95%CI: 0.35–0.54) were significant protective factors against early spontaneous abortion. This study further showed that the beneficial effect was mediated by increasing serum folate and reducing plasma Hcy levels in early pregnancy (98). However, Studies found that women with miscarriage had significantly lower serum folate (*p* < 0.001) levels (99), and folate deficiency showed no association with miscarriage (99, 100). More quantitative studies are needed to describe the relationship between circulating folate levels and miscarriage risk. Furthermore, the optimal dose and duration of FA supplementation for improving miscarriage outcomes warrant further investigation.

#### 2.4.2 MTHFR genetics modulation

Multiple studies confirm that MTHFR C677T gene polymorphisms are strongly associated with RSA. A study of 150 RSA patients and 120 controls linked the MTHFR 677TT genotype to reduced RBC folate concentration and associated the AG and GG genotypes of MTRR A66G with higher RSA risk, highlighting gene-nutrient interactions (101). A meta-analysis of 30 studies demonstrated that MTHFR C677T and A1298C (except AC vs. AA) gene polymorphisms were significantly associated with RSA risk in Asian populations, while MTRR A66G gene polymorphisms had limited impact (only in additive model: G vs. A) (18). The understanding of these genetic associations has guided clinical applications of FA supplementation. Individualized FA supplementation based on MTHFR or MTRR genotypes significantly reduced miscarriage rates compared to conventional or no supplementation (102). Moreover, HHcy reduced pregnancy rates in IVF or ICSI people, especially in women with MTHFR 677TT genotype, suggesting a genotype-specific metabolic vulnerability (103). Another study found that although no significant differences in miscarriage or live birth rates were observed among genotypes, those with the MTHFR 1298CC genotype had fewer oocytes retrieved, indicating the MTHFR polymorphism may influence ovarian response without affecting clinical pregnancy outcomes (104). Notably, a multiplicative interaction existed between serum folate deficiency in early pregnancy and maternal MTHFR 677TT genotype (OR = 3.50, 95%CI: 2.78–5.18) (98). Beyongd MTHFR, downregulation of MTHFD2 in villus tissues was found to disrupt folate-nucleotide metabolism, leading to the accumulation of 5-aminoimidazole-4-carboxamide ribonucleotide (AICAR) and subsequent inhibition of trophoblast invasion via AMP-activated protein kinase (AMPK)/Janus kinase (JAK)/Signal Transducer and Activator of Transcription (STAT)/Slug-mediated epithelial-mesenchymal transition (EMT) pathway, ultimately contributing to implantation failure in mice (105).

#### 2.4.3 Vitamin B12 modulation

While low vitamin B12 levels have been observed in women with miscarriage, the independent causal role remains unestablished. A study found that the combination of low B12 and high Hcy significantly increased early pregnancy loss (EPL) risk (OR = 4.98, *p* = 0.002) (100). Another case–control study of 186 Turkish women found that women with early miscarriage had significantly lower serum vitamin B12 (276 ± 135 vs. 320 ± 127 ng/L, *p* = 0.021) and serum folate (10.1 vs. 14.4 μg/L, *p* < 0.001) levels, and higher plasma Hcy (9.54 ± 4.28 vs. 6.24 ± 1.46 μmol/L, *p* < 0.001). However, logistic regression identified only elevated Hcy as a predictive factor for miscarriage, not low B12 or folate directly (99). Critically, a Mendelian randomization study found no significant causal relationship between genetically predicted vitamin B12 levels and spontaneous abortion and higher vitamin B12 showed a potential causal association with reduced risk of stillbirth (OR = 0.974, 95%CI: 0.953–0.996) (106). Furthermore, a Cochrane review of 5 RCTs (984 women) found vitamin B12 supplementation (5–250 μg/d) during pregnancy increased maternal and infant B12 levels (MD = +60.89 pmol/L and +71.89 pmol/L, respectively), but did not quantitatively report its effects on miscarriage (107).

To summarize, (1) Supplementation strategy: Periconceptional FA supplementation is a significant protective factor against early spontaneous abortion, primarily by increasing serum folate and reducing Hcy levels. The optimal dose and duration for miscarriage prevention require further investigation. (2) Circulating biomarkers: Lower folate status is strongly associated with RSA, though a direct link between folate deficiency and miscarriage remains unconfirmed. Elevated plasma Hcy is a more consistent predictive biomarker. (3) Genetic modulation: MTHFRC677T and A1298C gene polymorphisms are significantly associated with an increased risk of RSA, particularly in Asian populations. A strong multiplicative interaction exists between the MTHFR 677TT genotype and serum folate deficiency. (4) Nutritional modulation: While low vitamin B12 is observed in women with miscarriage, its independent causal role is not established.

### 2.5 Folate, MTHFR genetics, vitamin B12 and PTB

#### 2.5.1 Timing-, dose- and duration- dependent response of FA supplementation

Evidence regarding the relationship between FA supplementation and PTB risk is complex and inconsistent. Several large-scale cohort studies in China have consistently highlighted that pre-conceptional initiation of FA supplementation,is associated with a significant reduction in PTB risk and no benefit for post-conception initiation (8, 63, 108). A prospective cohort study of 240,954 women found that pre-conceptional FA supplementation was associated with 8% lower risk of PTB (RR = 0.92, 95%CI: 0.85–1.00, *p* = 0.040) (8). A retrospective cohort study of 201,477 women furtherly demonstrated that pre-conceptional FA supplementation significantly reduced PTB risk, with greater reduction when started ≥3 months before pregnancy (aOR = 0.80, 95%CI: 0.75–0.87) compared to 1–2 months (aOR = 0.85, 95%CI: 0.79–0.92) (108). However, other evidence points to post-conception benefit. A meta-analysis concluded that FA supplementation was associated with a significant reduction on the risk of PTD when initiated after pregnancy (RR = 0.68, 95%CI, 0.52–0.90), whereas no effect was found if the initiation time was before conception (84). Furthermore, a case–control study of 1,110 women (314 cases vs. 796 controls) in Jordan even indicated that FA supplementation during pregnancy was significantly associated with an increased risk of PTB (OR = 2.45, 95%CI: 1.33–4.52) (109).

Beyond timing, several factors significantly modify the association between FA supplementation and PTB risk, including compliance, chorionicity in multiple pregnancies, interpregnancys (IPIs) interval, and environmental exposures. In a study of 416 twin pregnancies, pre-conceptional FA supplementation was associated with a 0.385-week longer gestation and significantly reduced risks of PTB < 36 weeks (aOR = 0.519, 95%CI: 0.301–0.895). These protective effects were only significant with good compliance (≥4 times/week), among twins via assisted reproductive technology or dichorionic diamniotic twins (110). Additionally, insufficient folate intake could exacerbate the risk of PTB associated with non-optimal IPIs, as demonstrated by a study of 55,203 singleton pregnancies where short (< 6 months; OR = 1.76, 95%CI: 1.35–2.29) and long (≥120 months; OR = 1.65, 95%CI: 1.24–2.1) IPIs were linked to significantly higher PTB risk only in women with inadequate dietary folate and FA supplementation intake (111). Furthermore, pre-conceptional FA supplementation might also attenuate environmental risks. A national cohort of 1,229,556 Han Chinese primiparas found that each 10 μg/m^3^ increase in PM2.5 exposure was associated with a lower rise in PTB risk among women who initiated FA supplementation ≥3 months before pregnancy (HR = 1.09, 95%CI: 1.08–1.10) compared to non-users (HR = 1.12, 95%CI: 1.11–1.13, p-interaction < 0.001), demonstrating a protective effect of preconception FA supplementation against airborne particulate matter toxicity (112).

#### 2.5.2 Circulating folate levels

A prospective cohort study of 849 pregnant women in China, where the average serum folate level was high (11.95 ng/mL), reported a low overall PTB prevalence (3.76%) (89). Maternal serum folate deficiency was significantly associated with PTB (< 34 weeks) (aOR = 1.73, 95%CI: 1.27–2.36). Additionally, the risk of PTB was also higher among women who were of short stature (aOR = 1.83, 95%CI: 1.27–2.63), primiparous (aOR = 1.60, 95%CI = 1.15–2.22), and had exposure to passive smoking (aOR = 1.54, 95%CI: 1.02–2.31) (113). Evidence suggests that low circulating folate levels pose a greater risk for PTB in populations with folate deficiency. A hospital-based observational study in India found a significantly higher rate of PTB (16.94% vs. 7.53%) in the low plasma folate (< 8.6 ng/mL) group compared to women with sufficient folate levels (≥8.6 ng/mL) (114). Future research should prioritize large-scale quantitative studies on circulating folate levels to establish optimal FA supplementation dosages for diverse populations.

#### 2.5.3 MTHFR genetics modulation

In populations with adequate folate status, MTHFR C677T gene polymorphisms showed no significant association with the risk of PTB (115). This was supported by a prospective cohort study of 849 pregnant women in China, where the average serum folate level was high (11.95 ng/mL) (89). In contrast, under specific pathological conditions or functional folate deficiency, certain genetic variants were associated with an increased PTB risk. For instance, MTHFR C677T gene polymorphisms were associated with a significantly increased risk of PTB in HEV-IgM positive pregnant women, a link likely mediated by elevated Hcy leves (116). Additionally, women with the MTHFR 1298 AC genotype were at a higher risk for PTB (117). Beyond single variants, specific haplotype combinations and rare pathogenic variants also modulate PTB risk. MTHFR C677T, A1298C and MTR A2756G haplotype CAG (representing the C allele at MTHFR C677T, the Aallele at MTHFR A1298C, and the G allele at MTR A2756G) was protective for PTB (OR = 0.475, 95%CI: 0.233–0.970, *p* = 0.036), whereas the haplotype CCG (representing the C allele at MTHFR C677T, the C allele at MTHFR A1298C, and the G allele at MTR A2756G) increased the risk by 1.8-fold (OR = 1.81, 95%CI: 1.09–2.98, *p* = 0.018) (117). A rare pathogenic variant (c.1262 G > T) was strongly associated with PTB in a small Indian cohort (118). Paternal genotype may contribute as well. Paternal MTHFR 1298CC and MTHFD1 1958AA genotypes were associated with reduced risk for spontaneous PTB (20). A study of 191 infertile men undergoing ICSI treatment investigated paternal MTHFR C677T gene polymorphisms and showed that PTB rates among the different paternal genotype groups were 24% for CC, 14.63% for CT, and 9.67% for TT. However, these differences were not statistically significant (*p* = 0.35) (119).

#### 2.5.4 Vitamin B12 modulation

Evidence indicates that elevated Hcy, often linked to low vitamin B12, is a key mechanism in PTB. A case–control study demonstrated that placental stress caused by HEV infection increased Hcy due to alterations in maternal vitamin B12 levels and folate metabolism. This mechanism contributed to PTD and other adverse pregnancy outcomes in HEV-infected pregnant women, holding significant prognostic and therapeutic value (116). Supporting this, Bala et al. analyzed cord blood and placental tissue from PTB and term infants, revealing that PTB infants exhibited significantly higher cord blood Hcy levels (*p* = 0.020) and lower vitamin B12 (*p* = 0.005), with elevated oxidative stress (MDA, *p* = 0.04) and inflammatory markers (TNF-*α*, VEGF-A, MMP2/9). Their findings link fetal HHcy to placental inflammation and oxidative damage as potential mechanisms for PTB. However, the broader relationship between PTB and key co-factor nutrients in the folate metabolic pathway, such as vitamins B12 and B6, remains poorly defined due to limited research (120).

Taken together, (1) Supplementation strategy: The association is complex and inconsistent, with some studies indicating a reduced PTB risk with pre-conceptional initiation, while others show benefit only with post-conceptional initiation or even increased risk. Adherence, chorionicity, and environmental exposures (e.g., PM2.5) modify this relationship. (2) Circulating biomarkers: Lower serum folate levels are associated with an increased risk of PTB, particularly in populations with prevalent deficiency, though large-scale quantitative studies to define optimal ranges are lacking. (3) Genetic modulation: In populations with adequate folate status, MTHFR polymorphisms show no significant association with PTB. However, under conditions of functional folate deficiency, certain variants and haplotypes are linked to increased risk. Paternal genotype may also contribute. (4) Nutritional modulation: Elevated Hcy, often consequent to low vitamin B12 and impaired folate metabolism, is a key mechanistic link to PTB, associated with placental oxidative stress and inflammation.

## 3 Possible mechanisms

Folate, in its active forms, serves as a crucial donor of one-carbon units in cellular metabolism.

Dysregulation of folate metabolism can interfere with key biological processes such as amino acid metabolism, methylation reactions, and nucleotide synthesis, thereby impairing numerous physiological functions. Consequently, folate metabolic dysregulation may represent a common pathological basis for various adverse pregnancy outcomes, with the toxicity of Hcy serving as a central mechanism. HHcy, which can result from folate or vitamin B12 deficiency as well as the MTHFR 677TT genotype (121), was associated with an elevated risk of early pregnancy loss, placental complications such as PE and fetal growth restriction (FGR) (122). Furthermore, HHcy ia also linked to GDM (123) and PTB (122). Beyond the toxicity of Hcy, other plausible mechanisms include impaired folate-dependent nucleotide synthesis (105), aberrant DNA methylation (26), immune-mediated damage from FRAbs (27), and the direct anti-inflammatory effects of folate (28). Overall, adverse pregnancy outcomes may arise from multiple underlying backgrounds, such as MTHFR 677TT genetic predisposition and folate-vitamin B12 nutritional imbalance, and is driven by a complex interplay of multiple mechanisms.

### 3.1 Abortion

A meta-analysis of 23 studies involving 2,052 RSA cases and 1,476 healthy controls found that women with RSA had significantly higher Hcy levels both in plasma (SMD = 1.34, 95%CI: 0.76–1.93) and in serum (SMD = 1.46, 95%CI: 1.02–1.91) than controls, demonstrating a strong association between elevated Hcy and RSA risk (7). The pathogenic mechanism may involve direct damage to vascular endothelial function at the maternal-fetal interface and disruption of early embryonic development and trophoblast differentiation. Qi et al. observed elevated Hcy levels in women with RSA were correlated with increased endothelial microparticles (EMPs) and free plasma DNA. *In vitro* experiments demonstrated that Hcy exerted dose-dependent cytotoxicity on human umbilical vein endothelial cells (HUVECs), compromising membrane integrity and promoting EMP release. These findings indicated that elevated Hcy levels might contribute to miscarriage by inducing endothelial cell apoptosis, which leads to endothelial dysfunction and increases the release of endothelial microparticles (EMPs) and free DNA (124). From a developmental perspective, Capatina et al. investigated the influence of HHcy on early blastocyst development and trophoblast differentiation. They found that exposure of cultured blastocysts to high Hcy levels reduced cell number in the trophectoderm layer, most likely through increased apoptosis. Hcy also promoted differentiation of a trophoblast stem cell line. Both effects diminished the stem cell pool and were mediated in an endoplasmic reticulum (ER) unfolded protein response (UPRER)-dependent manner (122). Collectively, Hcy compromised embryo viability by inducing ER stress, leading to both trophoblast apoptosis and aberrant differentiation. Notably, the detrimental effects of HHcy may be mitigated by FA supplementation, which has been shown to improve pregnancy outcomes, potentially by reducing Hcy levels (97).

Beyond Hcy, deficiencies in key enzymes of folate metabolism and an immune-mediated mechanism involving FRAbs also play a role. A study identified MTHFD2 as a key regulator in RSA. Reduced MTHFD2 expression in villus tissues impaired folate-nucleotide metabolism, increased AICAR, and suppressed trophoblast invasion via AMPK/JAK/STAT/Slug-mediated EMT inhibition, leading to embryo implantation failure in mice (105). Although first identified in cerebral folate deficiency, FRAbs have also been implicated in miscarriage. FRAbs might disturb pregnancy establishment and maintenance by modulating trophoblastic biofunctions, placental development, decidualization, and decidua homeostasis as well as the functions of folate receptor beta positive (FOLR2+) macrophages (27).

Overall, dysfunction in folate metabolism is thought to contribute to RSA through multiple pathways, including the damaging effects of HHcy, impaired folate-nucleotide metabolism, and an immune-mediated pathway involving FRAbs. Elevated Hcy levels are considered to exert dose-dependent cytotoxicity on vascular endothelial cells at the maternal-fetal interface, which can promote apoptosis and disrupt vascular integrity. Concurrently, HHcy could impair early pregnancy by disrupting blastocyst development and depleting the trophectoderm cell pool via endoplasmic reticulum stress. Beyond Hcy, impaired folate metabolism is also implicated as a key factor. Deficiencies in key enzymes like MTHFD2 might disrupt nucleotide synthesis, thereby inhibiting trophoblast invasion. Furthermore, the immune-mediated pathway involving FRAbs is proposed to block cellular folate uptake, with the potential to directly interfere with trophoblast function, decidualization, and immune homeostasis.

### 3.2 HDP

Studies consistently showed that patients with HDP, especially those with PE, had significant HHcy. The HDP group (18.1 ± 6.2 μmol/L) had higher Hcy levels in serum compared with the control group (8.6 ± 3.9 μmol/L) (125). Moreover, Hcy levels exhibited a positive correlation with disease severity. Mean concentration of serum Hcy was higher (13.1 ± 6.4 μmol/L) in severe PE compared to mild cases (7.6 ± 2.8 μmol/L) (69). Receiver operating characteristic (ROC) analysis further demonstrated that the elevated Hcy levels in the first trimester effectively predicted PE, with cutoff values of >9.55 μmol/L (AUC = 0.859) (126) and >9.23 μmol/L (AUC = 0.978) (127). In addition, elevated Hcy concentration was a significant risk factor of fetal death in the PE group (128). HHcy is partly attributable to genetic factors, among which MTHFR gene polymorphisms was as a central risk factor. A low frequency of MTHFR 677\u00B0C allele (OR = 1.83, *p* = 0.040) was related to PE risks. Patients with MTHFR 677TT genotype had significantly higher Hcy levels than those with 677CC genotype (16). Furthermore, the elevated frequency of the MTHFR 677TT genotype was associated with a higher incidence of abnormal pregnancy, a link potentially mediated by elevated Hcy levels (125). In addition, MTR and MTRR gene polymorphisms also emerged as important modifiers (73).

On the other hand, folate deficiency can also lead to HHcy. Folate deficiency (< 4.25 μg/L) in placental tissue was correlated with HHcy and endothelial dysfunction in PE patients (*n* = 72) (69). A rat study demonstrated that FA deficiency elevated Hcy levels (*p* < 0.001), which might impair placental angiogenesis. However, key angiogenic markers, such as the soluble Fms-like tyrosine kinase-1 (sFlt-1)/ placental growth factor ratio (PLGF) ratio, remained unchanged in deficient models, suggesting alternative pathways independent of sFlt-1/PLGF ratio (72). Mechanistically, elevated Hcy concentration impairs vascular endothelial function, leading to reduced synthesis of nitric oxide (NO) and prostacyclin (PGI2) and an increase in thromboxane A2 (TXA2). This imbalance causes vasoconstriction, enhanced platelet aggregation, and arteriolar spasm, which disrupts utero-placental circulation, compromises fetal growth, and ultimately precipitates the characteristic pathophysiological changes of PE (25). However, the *in vivo* picture is complex. Animal studies using folate-deficient rat models showed no PE phenotype development despite elevated Hcy levels, although FGR occurred (72). These findings suggest that HHcy is an important contributing factor in PE, but may need to act in concert with other mechanisms to trigger the characteristic clinical manifestations.

Experimental and clinical evidence suggest that folate regulates trophoblast invasion primarily through DNA methylation, exhibiting a U-shaped dose–response relationship. *In vitro*, physiological FA (10^−7^ M) increased invasion by 2.4-fold in HTR-8/SVneo cells through reducing global methylation by 4.9% and DNA (Cytosine-5)-Methyltransferase 1/3A (DNMT1/3A) expression by 5-fold, while suppressing the methylation of tumor suppressor genes, Adenomatous Polyposis Coli (APC) by 4.1-fold (26). Conversely, excessive folate (>10^−4^ M) paradoxically decreased trophoblast invasion by 50% (26). Consistent with these findings, placental samples from PE pregnancies showed higher global methylation (39.6% vs. 36.1% in controls) and Long Interspersed Element-1 (LINE1) hypermethylation (+7%) (26), suggesting that aberrant methylation might underlie impaired spiral artery remodeling in PE.

*In vitro* studies in mouse and human endothelial cells have shown that treatment with 5-MTHF, but not FA, elevates tetrahydrobiopterin (BH4) levels, reduces superoxide production, and increases NO synthase activity. In primary endothelial cells isolated from women with hypertensive pregnancies, exposure to 5-MTHF, but not FA, restored the reduction in BH4 levels and NO synthase activity. *In vivo* studies in mice revealed that oral treatment with 5-MTHF, but not FA, prevented and treated hypertension in pregnancy when administered either before or during pregnancy, respectively (68). A study analyzed 3,001 mothers from the Boston Birth Cohort to assess the association between maternal folate status and placental maternal vascular malperfusion (MVM). Results showed that the lowest self-reported folate intake was associated with higher MVM risk (OR 1.45, 95%CI 1.03–2.05). Consistently, in a subset of 939 mothers with plasma folate measurements, folate insufficiency also increased MVM risk (OR = 1.65, 95%CI: 1.03–2.63). The combination of low folate and MVM correlated with the highest rates of PE (129). Collectively, these findings indicate that 5-MTHF confers direct endothelial protection through pathways independent of Hcy metabolism. Concurrently, maternal folate status was closely linked to MVM and the risk of PE, underscoring the critical importance of maintaining adequate folate levels for vascular health during pregnancy.

To summarize, folate’s role in HDP, especially PE, operates through three primary pathways. The classic HHcy pathway disrupts vascular balance, promoting vasoconstriction. Folate also regulates placental development via DNA methylation. Importantly, 5-MTHF, folate’s active form, directly protects endothelium by increasing BH4 bioavailability and NO production, independent of Hcy. Sufficient folate, particularly as 5-MTHF, is thus vital for adequate uteroplacental perfusion during pregnancy.

### 3.3 Fetal growth

Clinical studies confirm that elevated maternal Hcy levels during the periconceptional period or early pregnancy are an independent risk factor for impaired fetal growth. A prospective cohort study of 19,984 mother–child pairs in Shanghai found that higher maternal Hcy levels during periconception were associated with lower offspring birth weight (*β* = −2.30, 95% CI: −4.43, −0.16). Elevated early-pregnancy Hcy increased the risk of SGA infants (RR = 1.05, 95% CI: 1.01–1.08) (130). At 10–14 weeks of gestation, the concentration of Hcy was significantly higher in the FGR group than in the control group (19.65 μmol/L vs. 9.28 μmol/L, *p* < 0.0001). ROC analysis determined that the optimal first-trimester Hcy cut-off level for predicting FGR was >13.9 μmol/L (AUC = 0.788) (131). Animal studies using folate-deficient rat models (*n* = 18) also showed that FGR occurred with elevated Hcy (72). Elevated Hcy contributes to FGR via vascular endothelial dysfunction and placental insufficiency, similar to PE. Specifically, by inactivating nitric oxide, HHcy compromises endothelium-dependent vasodilation, leading to systemic vascular impairment. Concurrently, it promotes oxidative stress, trophoblast apoptosis, and thrombosis within the placenta, collectively disrupting perfusion and nutrient exchange, thereby restricting fetal development (25).

Thus, lowering Hcy levels may be as a core intervention strategy. Current research highlights two promising approaches with 5-MTHF or one-carbon metabolites (OCM) supplementation. *In vivo* studies in mice revealed that supplementation with 5-MTHF enhanced NO bioavailability through a key mechanism: elevating BH4 levels to restore NO synthase activity and reduce oxidative stress. This improved placental vasodilation and perfusion, thereby counteracting the vascular dysfunction that underlies FGR (68). A study in heifers investigated that nutrient restriction increased capillary area density (CAD) in intercotyledonary membranes and capillary number density in cotyledons (*p* < 0.10), whereas OCM (methionine, folate, choline, and vitamin B12) supplementation significantly increased CAD in endometrial glands (*p* = 0.01), indicating that OCM mitigates restriction-impaired vascular development (132).

Taken together, folate deficiency contributes to FGR primarily by inducing HHcy, which disrupts vascular endothelial function and placental perfusion through direct cytotoxic pathways.

Therefore, lowering Hcy may effectively counteract FGR pathogenesis. Effective strategies include 5-MTHF supplementation to enhance NO bioavailability and improve endothelial health, or OCM supplementation to directly increase placental vascularity.

### 3.4 GDM

Evidence indicates a significant association between high Hcy levels and the risk of GDM. Mendelian randomization (MR) analysis demonstrated a significant association between serum Hcy levels and GDM risk (OR = 1.28, 95%CI: 1.09–1.51, *p* = 0.003) (123). This finding is supported by an observational study, which found that increased Hcy levels in the second trimester were related to GDM (OR = 4.5, 95%CI: 1.5–13.0) (133). HHcy can result from folate or vitamin B12 deficiency as well as the MTHFR 677TT genotype (121). Clinical evidence supports that greater insulin resistance was significantly predicted by lower serum vitamin B12 (*β* = −0.622, *p* = 0.004) and higher RBC folate (*β* = 0.387, *p* = 0.018) (134). Specifically, a high folate-to-low B12 ratio can lead to a functional folate deficiency through the methyl-trap phenomenon, impairing the re-methylation of Hcy and regeneration of folates for DNA synthesis and repair. Consequently elevated Hcy concentration leads to endothelial dysfunction and oxidative stress (135). Separately, vitamin B12 deficiency independently impairs the conversion of methylmalonyl-CoA to succinyl-CoA, which is associated with insulin resistance that contribute to the etiology of GDM (135). Additionally, the MTHFR gene polymorphisms play a significant role in the pathophysiology of diabetes, including inflammation and insulin resistance (13). Beyond insulin resistance, another study indicated that FA-induced perturbations in one-carbon metabolism may affect β-cell function (2).

In summary, the association between elevated Hcy and GDM is attributable not only to its direct toxic effect on the endothelium and metabolism, but also to insulin resistance and β-cell dysfunction. These mechanisms are driven by disturbances in the folate-vitamin B12 metabolic axis and MTHFR genetic background.

### 3.5 PTB

HHcy, which results from dysregulated folate metabolism and altered vitamin B12 status, contributes to the risk of PTB (116). Multiple studies have identified that both high Hcy and low folate levels in early pregnancy are independently associated with PTB (136, 137). HHcy is thought to contribute to PTB by exacerbating vascular dysfunction and inducing placental insufficiency (25, 122). Placental folate transport may also play a key role. Folate Receptor 1 (FOLR1) mRNA expression was lower and protein concentration higher in PTB placentas relative to the control group (*p* < 0.05). Placental FOLR1 mRNA was positively associated with gestational age. Conversely, FOLR1 protein concentrations in placenta along with folate/vitamin B12 ratio in cord blood were negatively associated with gestational age (138). An epigenetic mechanism has been proposed to explain this dysregulation. The increased methylation of the FOLR1 gene in preterm placentas was associated with lower FOLR1 mRNA expression. This epigenetic silencing was further correlated with altered folate and vitamin B12 levels in cord blood, indicating that nutrient-dependent epigenetic regulation may contribute to PTB (139). Additionally, the anti-inflammatory properties of folate may reduce the risk of PTB. In a mouse model of lipopolysaccharide (LPS)-induced inflammation, pretreatment with FA (0.6, 3 or 15 mg/kg) prevented preterm delivery, fetal death, and intrauterine growth restriction (IUGR) by suppressing nuclear factor kappa-light-chain-enhancer of activated B cells (NF-κB) activation in both mouse placenta and human trophoblast cell line JEG-3, cyclooxygenase (COX)-2 upregulation in mouse placentas, and pro-inflammatory cytokines of interleukin (IL)-6 and keratinocyte-derived cytokine (KC) in amniotic fluid of mice. The findings suggest FA may reduce PTB risk through anti-inflammatory mechanisms (28). Clinically, higher maternal serum 5-MTHF and 5-CHO-THF levels were associated with a reduced anti-inflammatory cytokine profile in the cervix, whereas maternal folate levels were not associated with proinflammatory scores, supporting the role of folate in modulating local immune responses related to PTB risk (140).

In summary, folate helps maintain a successful pregnancy by regulating Hcy metabolism, ensuring placental nutrient transport, and exerting anti-inflammatory effects. Dysregulation in these pathways represents an important pathological mechanism underlying PTB.

## 4 Conclusion

The relationship between folate status and pregnancy outcomes is complex and non-linear. While FA supplementation is unequivocally beneficial for preventing neural tube defects, its effects on other outcomes like GDM, HDP, and PTB are highly dependent on dose, timing, and individual patient factors. FA supplementation may exhibit a U-shaped association with pregnancy outcomes. Moderate-dose supplementation often shows protective effects, whereas no supplementation, low-dose, short-term, or inappropriate high-dose use can be ineffective or even increase risks. The influence of folate is further modified by an individual’s genetic background (particularly MTHFR genotype) and vitamin B12 status, with imbalances in the folate-B12 axis being a significant risk factor for several adverse outcomes. The toxicity of Hcy serves as a common pathological link across many of these conditions.

However, critical problems remain unresolved. The foremost issue is the lack of definitive, quantitative thresholds for circulating folate levels (in RBC or serum) that are associated with optimal outcomes for specific conditions beyond NTDs. The existing evidence is largely based on supplementation dosage, not achieved physiological levels, creating uncertainty for clinical practice. It is also unclear how to best personalize supplementation in the context of genetic variations and varying baseline nutritional status to achieve a target protective folate range.

Future research must prioritize longitudinal and large-scale studies to establish the quantitative relationship between circulating folate concentrations and the risks of GDM, HDP, PTB, and impaired fetal growth. This should be followed by RCTs targeting trimester-specific optimal folate ranges, including genotype-stratified trials and standardized measurement of specific folate forms (e.g., UMFA, 5-MTHF, 5-CHO-THF, THF). Mechanistic research should continue to clarify the roles of specific folate metabolites, epigenetic regulation, and immune pathways in different pregnancy complications.

The primary clinical guidance emerging from this review is the necessity to move beyond a universal supplementation recommendation. The ultimate goal is to implement a precision nutrition framework where FA supplementation is personalized. This would be guided by the objective of achieving and maintaining an individual’s circulating folate within an optimal, evidence-based range, determined through pre-conception or early-pregnancy screening that considers genetic and nutritional biomarkers. The critical next step is to define this target range, transforming pregnancy nutrition from a population-based recommendation to an individualized, biomarker-driven preventive strategy.
